# Exodontia skills acquisition: Focusing on clinical teaching and training

**DOI:** 10.1371/journal.pone.0286737

**Published:** 2023-06-07

**Authors:** Nashreen Behardien, Priscilla Brijlal, Nicolette Vanessa Roman

**Affiliations:** 1 Department of Maxillofacial and Oral Surgery, Faculty of Dentistry, University of the Western Cape, Cape Town, South Africa; 2 Department of Oral Hygiene, Faculty of Dentistry, University of the Western Cape, Cape Town, South Africa; 3 South African Research Chair in Family Studies, Centre for Interdisciplinary Studies of Children, Families and Society, Faculty of Community and Health Science, University of the Western Cape, Cape Town, South Africa; Ankara University Faculty of Medicine: Ankara Universitesi Tip Fakultesi, TURKEY

## Abstract

**Objectives:**

The aim of the research was to evaluate the traditional exodontia block course. The objectives were to explore the experiences and views of students, clinical teachers and dental practitioners of the various elements of the course curriculum.

**Methods:**

The study was a qualitative, participatory action research study using descriptive analysis. The study was conducted at a Dental Faculty in South Africa. A purposive sample of students, clinical teachers and dental practitioners were invited to participate. Focus group discussions were used to collect data which was analysed by an external coder.

**Results:**

The study population consisted of 15 undergraduate dentistry students, 10 clinical teachers and seven dental practitioners. Four broad themes with sub-themes emerged from the study. The main themes identified strengths and deficiencies of the traditional course and made recommendations for its improvement. The themes identified were i) Integration of knowledge and skills, ii) Block course structure, iii) Challenges associated, and iv) Recommendations for improvement. Overall, the participants were satisfied that the course met its objectives. The results pertaining to clinical skills acquisition identified that teaching the use of elevators and luxators in the course, and standardisation of terminology among all clinical teachers as areas requiring attention. Teaching and learning strategies such as community-based learning, peer learning, case reviews, feedback and visual technology were viewed by the student, as well as clinical teacher samples, as strategies most beneficial to clinical learning.

**Conclusions:**

The review of the curriculum for exodontia skills acquisition and development, provided several benefits. Firstly, this research served as a quality assurance indicator. It further highlighted many teaching and learning strategies that would improve clinical skills development, reduce stress and anxiety, and support student learning. To a large extent, pertinent information was obtained that served to inform the subsequent redesigning of the course. The findings of the study augment the literature currently available on the best practice for exodontia skills acquisition and development and provide baseline information for the planning and redesign of related courses.

## Introduction

Tooth extraction [exodontia] is a commonly performed dental procedure worldwide [1, 2]. Primarily, the procedure is conducted for the treatment of pain and sepsis, for space creation (orthodontics), for the prevention of osteoradionecrosis and for aesthetic reasons. Dentistry academic institutions are responsible for training dentists who are competent in this basic procedure [3]. Although very commonly practised in South Africa, as evidenced by the number of missing teeth [4], very little research on best practices for teaching and learning the skill of simple [forceps] tooth extraction exists. Due to the invasive nature of the procedure and risk of severe complications, it is important that dental students receive sound instruction in the fundamentals of oral surgery in order to become competent clinicians [5] as this would have significant bearing on patient outcomes. A reduction of associated complications [6] would reduce patient morbidity and the cost of corrective surgery and possible litigation.

Competence has been defined as a measure of performance that is the active, behavioural expression of expertise lying on a continuum from novice to expert [7]. The journey to competence begins with the beginner, the unconscious incompetent, and culminates in the consciously competent individual [8]. For disciplines such as dentistry, clinical teaching and training is core to developing proficiency or competence. Historically, the Halstedian model was employed as a strategy to facilitate the transition of students from the novice to competent. This model has for the most part been replaced by competency-based education [9]. A variety of factors that influence the development of clinical competence, have been identified in the literature [10, 11]. A study on undergraduate medical students in Saudi Arabia [10] reported that factors such as the authenticity of the learning experience, the organisation of the teaching activities, supervisor factors, and patient case-related factors play a role in student clinical learning, while a study on medical students from a UK university reported that some students in their sample learnt from clinical experience, whilst others learnt from hands-on experience only [11].

**Fig 1:**
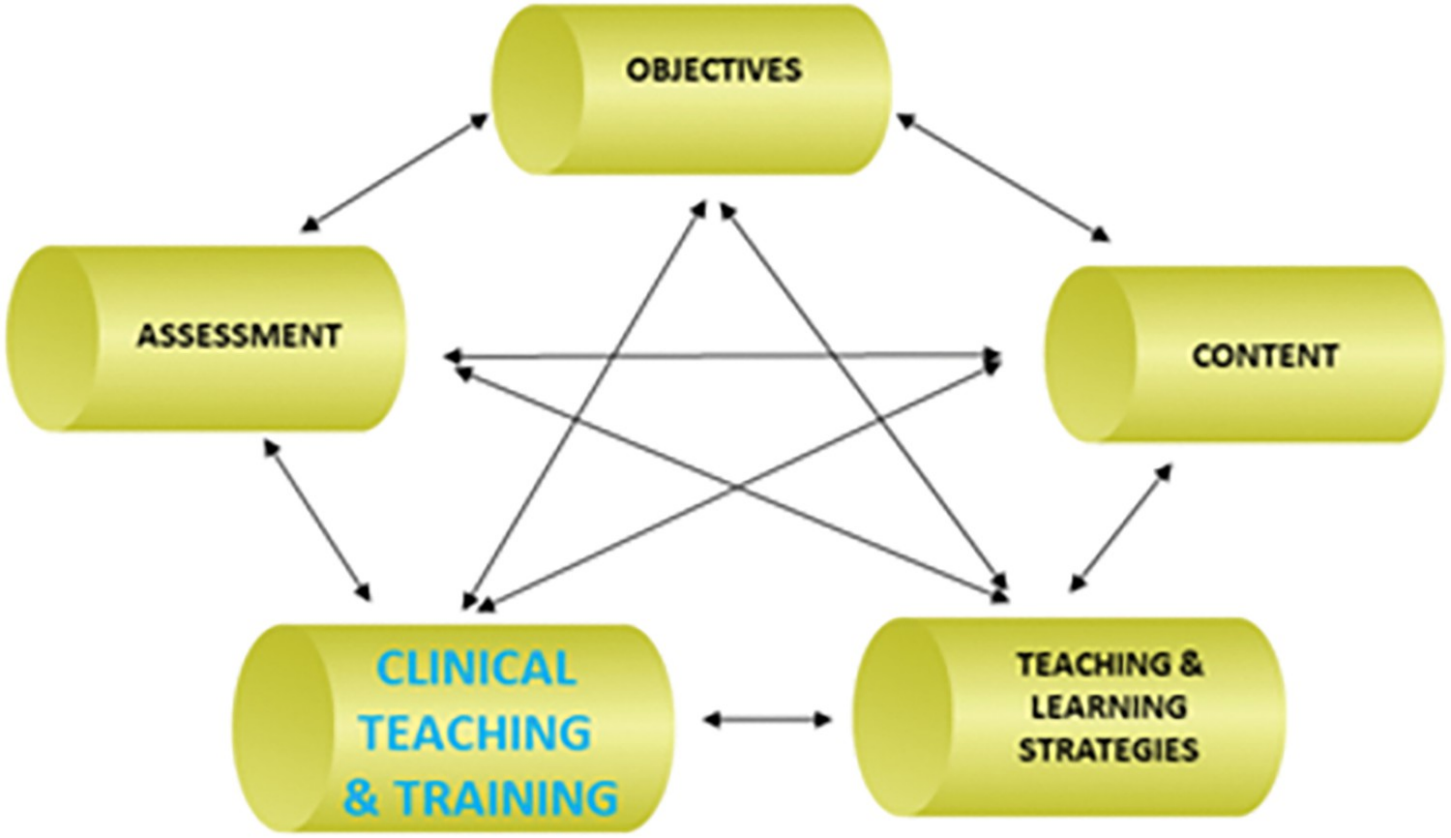
Elements of the curriculum applicable to this research.

Clinical teaching and training, an inherent feature of health professions curriculum (Fig 1) enables students to provide treatment on patients in a real-world setting. Quality assurance of the procedures carried out by student clinicians is supervised by clinical teachers (CTs), also known as supervisors or preceptors [12]. Clinical training is a critical part of the curriculum and is vital in shaping the basic skills and professional capabilities of students [13, 14]. Clinical training provides the opportunity for students to link their theoretical information with scientific facts [15] and to gain experience gradually through treating patients. A number of clinical training models exist and include service learning, apprenticeship, and work-integrated learning (WIL). These contribute to the development of students in the clinical context and demonstrate the importance of experiential learning in clinical skills development [16, 17].

The commencement of acquiring a new clinical skill often starts in a simulated clinic or a skills laboratory; recognised as preclinical teaching and learning. The preclinical environment may include demonstrations and practice on bench models or simulators. Once a level of readiness has been achieved by the student, he/she may progress to the next level, that of training on the clinical platform. In this study, the ‘service learning’ model most accurately resembles the clinical training aspect experienced by the students of the programme in this study. By definition, service learning combines educational goals with service to the community whereby the community and the training institution are equal partners. The three main goals of service learning are improving learning, promoting civic engagement, and strengthening communities [18]. Although the treatment of patients in a real-world environment provides the most appropriate learning environment for dentistry students to integrate their knowledge of basic dental science and operative dental technique skills [19], it is associated with an increased risk of poor patient outcomes [20]. This is usually mitigated by direct clinical supervision by experienced dental professionals [21]. In this study, the terms ‘clinical teacher’ and ‘supervisor’ are used interchangeably and refers to the dental practitioner who oversees the clinical treatment carried out by students in the clinical setting. Clinical teachers form an integral part of the clinical teaching and training process and their role is well established in the literature [22–24]. The skills contributing to effective clinical teaching include the skilful guiding of students, the ability to bridge theory and practice and initiate appropriate changes in knowledge, communication, technical skills, and attitudes and behaviours in daily practice for the benefit of patients and communities [18, 25]. Ultimately, clinical teaching or supervision involves maximising the learning opportunities of the student clinicians while keeping both the student and the patient free from harm [23].

For this research, the exodontia curriculum with its various facets is reviewed. Detailed information on the topic is sparse. The existing literature largely focuses on content delivery and assessing competence [5, 26–30] and not clinical skills development. In literature from the United Kingdom (UK), significant variations in the curricula among its dental schools are apparent. The majority use a lecture programme to deliver oral surgery teaching, with some integrating small group tutorials [31]. European dental schools have also shown considerable variation in dental curricula [32]. Essentially, the didactic teaching occurs approximately in years two and three, with the clinical procedure starting in year three. Moreover, the teaching and learning strategies vary among institutions, with some making use of phantom heads to teach the clinical procedure before progressing to patients and others making use of cadavers to introduce the clinical procedure. Clinical assessment practices also vary in addition to variation in measurements of proficiency and quota requirements [33].

In this research, at a dentistry school in South Africa, a learning unit in a Maxillofacial and Oral Surgery module focusing on the teaching and learning of tooth removal (exodontia) was evaluated. The Exodontia Block Course is presented in a three-and-a-half-day (3.8) contact session in the third year of study. Due to the traditional block course not having been systematically reviewed for an extended period, the module coordinator (the primary researcher) decided to review the course and redesign it by incorporating a teaching and learning strategy (deliberate practice) aimed at improving the procedural skill of tooth extraction. As part of a larger study evaluating the traditional exodontia block course, this paper aims to describe the experiences and perceptions of CTs, dentistry students, and dentists in practice of the preclinical component of the tooth extraction procedure with emphasis on the strategies employed, the instruments or equipment used, and the logistics of the course. Furthermore, the challenges and recommendations for improvement of both the preclinical component and the clinical training aspect are reported.

## Methods

### Study design

This was a qualitative study using descriptive analysis. Participatory action research (PAR) was used as the research methodology and appreciative inquiry (AI) as the framework for data collection and analysis.

#### Participants

The research was conducted at a dentistry school in South Africa. A purposive sample consisted of undergraduate dentistry students, CTs and dental practitioners (DPs). Only students who had completed the Exodontia Block Course, CTs who taught in the Maxillofacial and Oral Surgery Department, and DPs who completed the course at the institution as undergraduate students were eligible to participate. Invitations to participate in the research were sent to the 3^rd^, 4^th^ and 5^th^ year students via the class representatives and through the ‘announcement’ tool of the learning management site. The DP sample were general dentists from various private and public settings. These participants were recruited using a snowballing approach through contacts of the primary investigator (PI). Information pertaining to the nature of the research was shared with participants, and consent for the research was collected via a Google Form.

#### Ethical considerations

The Biomedical Research Ethics Committee of the institution granted ethics approval (BM19/10/23). Focus group confidentiality binding forms were completed by all participants. The research assistants involved in the research where reminded of the protection of data act. Data were stored on Google Drive and a password-protected laptop. The participants were de-identified by replacing their names with a participant identifier thus ensuring anonymity. Participation was voluntary and no monetary or other incentives were provided.

#### Data collection

**Fig 2:**
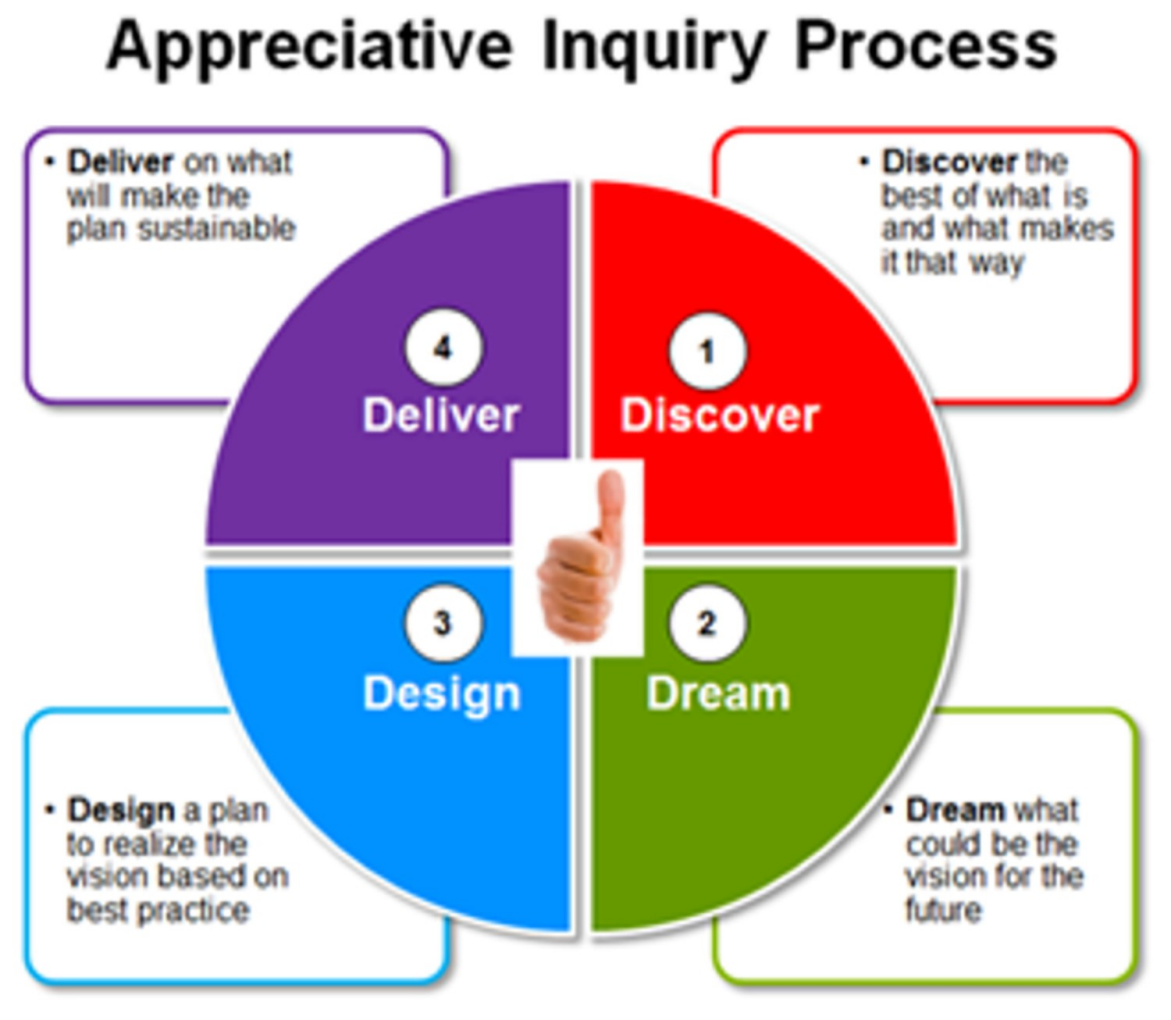
4-D model of the appreciative inquiry framework. Available from: https://blog.readytomanage.com/is-appreciative-inquiry-a-useful-workplace-tool/.

Data collection commenced in July 2020 and was completed in March 2021. A Google Form was sent to participants to collect demographic information. The PI conducted the first focus group discussions (FDGs), initially with a group of two students and in a separate session, with a group of six CTs. These FGDs were conducted in-person (face-to-face) and constituted the pilot study. As a means of eliminating any measure of bias, an external facilitator was used thereafter to conduct the remaining FGDs. Because of the COVID-19 pandemic, these FGDs were conducted online using the Zoom and Google Meet platforms. Data from the pilot study were added to the study data and included in the analysis since there were no significant differences. A semi-structured interview guide informed by the AI framework (Table 1) was used flexibly with questions exploring participants’ experiences of the block course. Appreciative inquiry [34] is considered both a theory of organising and a method for changing organisations and communities and shares some of the core principles of the action research methodology. The principles that guided the research [35] are expressed as the 4-D AI model (Fig 2). The 4-D model refers to four phases, namely Discovery, Dream, Design, and Destiny or Delivery. The Discovery phase is based on the best of what currently exists [36]. The Dream phase reflects a vision and logic to identify the ideals of what might be, while the Design phase consists of a collaborative exercise and agreement of what should be [36]. Lastly, the Delivery or Destiny phase refers to a collective experimentation to discover what is possible [36].

**Table 1:** Semi-structured interview guide using the AI framework–Examples of the questions used.

**Phases of Appreciative Inquiry**	**Question Guide**
	Inquiry into the experiences of the participants regarding the following: • Their individual experiences on the various components of the block course • An in-depth inquiry into the specific components of the course ○ Individuals’ peak experiences of the course ○ Suggestions for course improvements Questions pertaining to the various teaching strategies • Their main wish for an ‘ideal’ block course
Discovery	Which parts of the block course did you think worked well? Do you think the demonstrations were valuable for those types of clinical activities? How do you feel about the assessments (80% mark to progress to patient treatment; clinical procedures)
Dream	If you could have one wish regarding the course, what would it be?
Design	Inquiry into perceptions of teaching and learning strategies, assessments, pace, layout (scaffolding of theory and practical), etc. of the course. The facilitator used participant responses as a foundation for further probing.
Destiny/Delivery	How would you like to see the course presented in future?

Focus groups were used as a data collection instrument because it allowed for the collection of rich data and a reduction in potential researcher bias [37]. These discussions varied in duration but ultimately ceased when no new information or repetition of responses occurred (data saturation). For the student sample, four students were in their third year of study, four students were in their fourth year of study, and seven students were in their fifth year of study. The time between the completion of the traditional block course and the interview differed among the various student year groups. The ‘fifth-year’ group had graduated and had spent approximately two weeks performing mandatory community service in the public sector when the FGD took place. The time between completion of the course and the fifth-year group discussion was approximately 24 months for the pilot student group and 29 months for the fifth-year group. For the third- and fourth-year groups, the time interval was approximately one and 22 months, respectively. The discussion format for collecting data increased the reliability of the data, particularly in instances in which there was a longer time interval. The effects of recall loss are mitigated by the group discussion format since a benefit of this format is that it allows for the triggering of memory.

#### Analysis

The recordings were transcribed verbatim and ‘cleaned’ for better flow. Filler words such as ‘the’ and ‘so’ were removed. A research assistance was employed to do the transcriptions, and the data analysis was conducted by an independent coder and analyst who was familiar with qualitative data. After preparing the raw data for analysis, Tesch’s eight steps for analysing data was employed. The eight steps were i) gaining a sense of the whole by reading through all the transcribed interviews, ii) searching for underlying meanings, iii) compiling a list of all the topics and grouping them together into clusters, iv) abbreviating the topics in view of data coding, v) transforming the topics into themes and reducing the list of themes, vi) deciding on the themes and alphabetising the final themes, vii) performing a preliminary analysis, and viii) recoding existing data where required [38]. Only the results pertaining to the focus of this paper, that of preclinical, and clinical teaching and training, are presented in this paper.

#### Rigour

The inclusion of various stakeholders in the research in addition to member checking improved the trustworthiness of the outcomes of the research. The researcher was aware of her role within the research and the potential for bias stemming from her experience in the field. The researcher endeavoured to remain reflexive throughout the research through debriefing sessions with one of her supervisors.

### Findings

#### Participants

The participants consisted of 15 undergraduate students, 10 CTs, and 7 DPs. An overview of their characteristics and demographic information is presented in Table 2. Of the 261 students invited to participate in the research, 15 (5.75%) acceded. Although this constituted merely 6% of eligible students, the qualitative nature of the research contributed to ensuring reliable data. The focus group discussions were conducted until data saturation was reached. A possible reason for the low participation rate could be the full academic programme of the students and lack of free time. The FGDs took place after hours as requested by the participants. In addition to this, the research took place during the COVID-19 pandemic. This period was marred by uncertainty in all aspects of life, which could have contributed to the low participation. The ages of the participants in the student sample ranged from 22 years to 25 years, with a mean age of 22.87 years. The 12 CTs who were eligible to participate in the research were invited, and 10 responded positively (83.3%). The ages of the participants in the CT sample ranged from 27 years to 62 years, with a mean of 36.6 years. The number of years of experience at the time of the research ranged from 4 years to 30 years. The broad range in age reflected a good blend of content experience as evident in a response from a student: *I mean*, *supervisors know their stuff*. *We just don’t necessarily grasp it the minute they give it to us (SIV3)*, and a group of younger CTs who were able to identify with the current generation of students [39]. A group of CTs of a similar generation may be able to establish a good rapport with the student population thus improving knowledge transfer as reflected by SIV3:

*[S]upervisors*, *obviously they’ve been doing it*, *they’ve got years of experience*. *but we don’t learn the same—and they teach us*, *they’ll teach us once*, *like*, *they’ll cover a lecture and everyone is listening*, *but we don’t learn the same*. *So then you’ll go to someone else in your class*, *who will kind of demonstrate or teach you*, *or just rephrase what the supervisor said in their way*, *or their explanation makes more sense to you*. *Because*, *I mean*, *this is someone who’s your age*. *This is someone who processes similar to you*.

**Table 2:** Demographic information of the participants.

**Student Sample (N = 15)**									
**Pseudonym and Participant Identifier**	**Characteristics**	**M**	**F**	**Age Range in Years**					
	Entire / Full sample	8	7	22–25					
	Year 3	1	3	22–24	N = 4				
	Year 4	2	2	22–23	N = 5				
	Year 5	5	2	23–25	N = 7				
								
Jupiter1 –SV7	5	x		23					
Skye–SV6	5		x	25					
Mars–SIV1	4	x		22					
Dandelion–SIV2	4	x		22					
Buttercup–SIV3	4		x	23					
Rose–SIV4	4		x	23					
Jupiter2 –SV1	5		x	24					
Panda Bear–SV2	5		x	24					
Sunflower–SIII1	3		x	22					
Saturn–SIII2	3	x		22					
Owl–SIII3	3		x	20					
Cleveland–SIII4	3	x		24					
Dolphin–SV3	5		x	23					
Lion–SV4	5	x		23					
Anemone–SV5	5		x	23					
**Clinical Teacher Sample (N = 10)**									
**Pseudonym and Participant identifier**	**Characteristics**	**M**	**F**	**Age in Years**	**Number of Years Qualified**	**Additional Qualification**	**Hours p/w Practising as a GDP**	**Hours p/w Teaching**	**Years as a Clinical Teacher**
		4	6						
Burbae–CT1	Private practitioner / Academic		X	37	15	PGDip	15	20	10
Mars–CT2	Private practitioner	X		35	12	PGDip	30	8	1.5
Appa–CT3	Private practitioner		X	36	13	-	40	20	1
Lilly–CT4	Private practitioner		X	35	12	PGDip	28	12	4.5
Kitty–CT5	Private practitioner		X	35	13	PGDip	30	16	5.5
Jazz–CT6	Private practitioner	X		62	30	Oral hygiene; LLB; Hon Oral Surgery	30	20	30
Constantia Nek	Private practitioner	X		36	14	MSc(dent)	50	10	7
Lion	Private practitioner	X		27	4	-	48	8	2
Daisy	Public sector		X	32	9	PGDip	40	10–12	4
SI	Specialist in training	X		31	9	MSc(dent)	20	10	4
**Dental Practitioner (DP) Sample (N = 7)**									
**Pseudonym and Participant identifier**	**Characteristics**	**M**	**F**	**Age in Years**		**Additional Qualification**			
		4	3	23–33					
Sirius–DP1	Private practice	x		28	5	PGDip			
Rose–DP2	Academic facility		x	26	3	PGDip			
Mango–DP3	Private practice; Public dental clinic	x		33	9				
Daisy–DP4	SANDF		x	31	8				
Peony–DP5	Private practice; Public dental clinic		x	28	4	PGDip Aesthetics			
Apple–DP6	Public dental clinic	x		23	1	-			
Lexus–DP7	Private practice; Public dental clinic	x		27	4	-			

Key: GDP–general dental practitioner; PGDip–postgraduate diploma in dentistry; p/w–per week; SANDF − South African National Defence Force

Clinical teachers were predominantly part-time faculty, primarily from the private sector, who spent between 8 hours and 20 hours per week teaching on the clinical platform and a mean of 33.1 hours per week practising as general dentists. In addition, 80% of the CTs held postgraduate qualifications, which included an honours degree in oral surgery, master’s degrees in dentistry, postgraduate diplomas in oral surgery, and a law degree. The two faculty members who did not have a postgraduate qualification were enrolled for a postgraduate diploma in oral surgery at the time of the research. Furthermore, three of the participants were no longer employed at the school at the time of the research. One participant had emigrated, one had moved to the public sector, and the remaining participant had started training as a specialist maxillofacial surgeon.

Of the DP sample, all participants were employed in private practice, the public sector, an academic institution, or the National Defence Force. The age range was between 23 years and 33 years (mean age: 28 years). The time from qualification to the current research was between one year and nine years. Demographic information and characteristics of these samples are presented in Table 2.

#### Curriculum

Four broad themes with sub-themes were identified by the independent coder and analyst. These themes were finalised after a discussion between the principle investigator (NB) and the coder. The main themes identified the strengths, deficiencies, and recommendations for improvement of the traditional block course as experienced by the participants of the research (Table 3). The themes were i) *Integration of knowledge and skills;* ii) *Block course structure*; iii) *Challenges associated*, and iv) *Recommendations for improvement*. The first two themes reflected the strengths of the course. The themes and sub-themes that are described and discussed in this paper relate primarily to clinical teaching and training or clinical skills development. The four phases of the AI framework are used to present these findings (Table 4). These phases are Discovery, Dream, Design, and Destiny.

**Table 3:** Master Table presenting the themes and sub-themes that emerged from the research.

THEMES	SUB-THEMES
1. Integration of knowledge and skills	The existing exodontia block course provides a foundation for oral surgery practice. The layout (components) of the existing exodontia block course is well planned.
2. Block course structure	Didactic teaching is supplemented by clinical exposure. 2.2 Clinical teachers assist with consolidating theory and practice. Teaching and learning strategies support learning. Assessment for and of learning Activity workbooks Demonstrations and videos 2.4 Student confidence
3. Challenges associated	3.1 Block course duration 3.2 Clinical teaching and training 3.2.1 Patient and operator positioning Covid-19-specific challenges Clinical rotation group size 3.3 Student stress and anxiety 3.4 Reliance on teachers 3.4.1 Ownership of learning 3.4.2 Clinical teacher support for managing medical emergencies
4. Recommendations for improvement	4.1 Students’ recommendations to improve teaching the skill of tooth extraction 4.1.1 T&L strategies • Visual technology and demonstrations • Simulated patients • Case reviews • Debriefing • Peer learning • Community-based learning • Feedback • Mandatory assessment • Patient and operator positioning 4.1.2 Curriculum issues • Use of elevators • Earlier introduction to exodontia • Standardising the content and terminology 4.2 Clinical teachers’ recommendations to improve teaching the skill of tooth extraction 4.2.1 T&L strategies • Scaffolding • Community-based learning • Peer learning • Visual technology • Pre-block orientation • Patient and operator positioning • Skills laboratory 4.2.2 Curriculum issues • Standardising the content and terminology • Additional practical assessment • educing the didactic teaching

**Table 4:** Themes, sub-themes, and supporting quotations on clinical skills curriculum viewed through the lens of appreciative inquiry.

**DISCOVERY–*Appreciating* w*hat works well***		
**Theme**	**Sub-Theme**	**Quotation (Response)**
Integration of knowledge and skills	The traditional block course provides a basic foundation for oral surgery practice	• So, it’s laying down the foundation phase of, I guess, your oral surgery practices. (DP2) • [W]hat I can say is that the block course did equip me with the necessary knowledge and skills to confidently give local anaesthetics and to do extractions. (DP4) • It’s not to say you have done the block course; you’re going to do everything perfectly, but I think it gives you that foundation. (CT8)
Block course structure	Didactic teaching is supplemented by clinical exposure	• In this block, we had a lecture in the morning and then also went into clinics on the same day, actually immediately after. So everything was kind of fresh. And you could really try and apply your knowledge more as opposed to learning something prior. (SIII3)
	Clinical teachers assist with consolidating theory and practice	• The supervisors [CTs] work really well to get us where we need to be. (SIV4) • Another part of it that I enjoyed was [that] we did have full support from our supervisors and they were very patient with us. (DP5)
	Student confidence	• I would say though that [the] majority of the learning does come after the block course is done, but I think that goes without saying. Once you actually get a chance to practise over and over again, the extraction component, then you kind of get a hang of it and understand what or understand better what was done in the block course. (DP2)
**DREAM *(Envisioning Results)***		
**Theme**	**Sub-Theme**	**Quotations (Response)**
Recommendations for improvement	Students’ recommendations to improve strategies for teaching the skill of tooth extraction: Curriculum issues– ✓ Earlier introduction to exodontia ✓ The use of elevators	*Curriculum issues–Earlier introduction to exodontia* • If they can just move it to the beginning of third year, Feb or whatever. I mean, imagine students having Oral Surgery from beginning third year. By the time you get to fourth year and final year, you’ve nailed so many things on such a good level—you know, skill level. … if we had this block from the beginning of third year, you’ve had two years’ university experience … Basically, by the time we get to final year, you don’t really need a supervisor; they’re just supervising and checking tiny things. I just feel like it’s a bit too … late in third year. (SIV4) *Curriculum issues–the use of elevators* • There was just one thing that I would have liked. … is the use of elevators. So in our block, we just use the normal forceps and then at a later stage, we get to learn how to use the elevators. But I feel that the students need to be taught from the get go. Just like you get comfortable with the forceps, you should get comfortable using an elevator. (SIV4)
	Students’ recommendations to improve strategies for teaching the skill of tooth extraction: Teaching and Learning strategies - ✓ Debriefing ✓ Skills laboratory	*Teaching and Learning Strategies—Debriefing* • I think a debriefing session is really good. Good idea at the end of the session because anything could happen in the clinic. And anyone can learn from it, like how to manage and what was done. So it’s like a learning point for everyone from the session. It’s not just something that happened in a cubicle and someone must be traumatised over it. It’s like everyone learns from it. (SV3) *Teaching and Learning Strategies–Skills laboratory* • [E]levate the demonstration. (SIII4) • I don’t know if this is realistic, if this exists in the world, but like working on a dummy [mannequin]. The mouth structure is difficult because patients differ. Obviously, the oral cavity is different. But your basic structures are there. You have your external oblique ridge—it’s there. So to have like a dummy kind of figure thing, so that when you see a patient, you can sort of gauge what you’re supposed to look for. And that you inject doing the dummy thing. I would have done the dummy thing because, yeah, taking into consideration the fear that students have. (SIV) • I think that [a skills lab] would, that could work as, as opposed to just going directly to a patient, but just for your positioning because that is a major problem when you start clinics. So, for that, and for instruments selection, I think it would be beneficial to practise first. (SV6)
	Clinical teachers’ recommendations to improve teaching the skill of tooth extraction: Teaching and Learning strategies - Skills laboratory	• What about having a skills lab? Somewhere where the students can go afterwards [after hours]. Where there are the mannequins and typodonts and everything. Where they can go in their own time, maybe in a group of friends … where you can go and do the things after hours. (CT9) • The skills lab is a brilliant idea. … Tygerberg has … a sort of skills lab. … [M]aybe it just needs to have more resources put into it—some more videos that are automatically there that students can click onto if they wanted to learn how to do an upper molar extraction. That there’s a typodont with the correct forceps for the procedure and a video that they can just click on [to] show them how to engage the forceps. (CT7)
**DESIGN *(Co-constructing)***		
**Theme**	**Sub-Theme**	**Quotations (Responses)**
Challenges associated	Clinical teaching and training ✓ Clinical rotation groups ✓ Patient and operator positioning	*Clinical rotation groups* • [W]e were a smaller group, split in half. And it just has much more of an impact if it’s one supervisor speaking to five students than it would be to one supervisor speaking to 20 or 10 students. Because I’m a very shy person, like especially when it comes to school stuff, I’ll pull the supervisor aside to ask questions because I don’t want to feel like I’m asking dumb questions. So if there’s five of us, I’d be more free to ask, and it just encourages interaction also. (SIV3) • I don’t think I learnt a lot from it because it’s really hard to see. There were about 10 to 12 of us in one group, and obviously, the mouth is such a small area that we’re placed around him to kind of engage and see. (SIII1) • The quality that you give will be better when you have a smaller group. (CT1)
	Block course duration	• The cases are plenty; there’s more than enough. But other resources are limited in terms of chairs. As [for] the sessions—everything is rushed. The clinical teachers—you can’t spend half an hour to an hour with one student to teach them because you’ve got 10 other students that are waiting for you. (CT10) • The third-year students are very fresh. So you basically hold their hand the whole way from beginning to end, as soon as they walk into the cubicle until they leave. So, and that makes it difficult when it’s a big class because they need the individual attention. … So time is definitely an issue. … We have the knowledge and the skill; we just don’t have the time. (CT8) • … dedicate more time to students being in clinical setting. (DP1) • I think Oral Surgery should be two weeks, over a period of two weeks. We can do lectures, yes—100% in the morning and then the clinical exposure for a whole week rather than coming in in the morning and sitting. Then you’d be there at eight and you start with patients at eight. (SIV4)
	Student stress and anxiety	So what I found is that with students coming into the clinic for the first time, they may obviously not remember how to hold an instrument or whatever it is. And so instead of teaching them, you criticise and we assess. And so we don’t actually allow the student to improve and to learn. So I think that a decrease in patient numbers may be something to look at because Oral Surgery becomes more service rendering. (CT1) Now you don’t know which information to use for what and then that kind of does put a lot of pressure on you because now you have an actual person sitting in front of you in pain, and you need to do something about it. (SV6)
Recommendations for improvement	Students’ recommendations to improve teaching the skill of tooth extraction: Teaching and learning strategies - ✓ Community-based learning ✓ Peer learning ✓ Case reviews ✓ Feedback ✓ Visual technology	*Community-based learning* • The outreach to Kraaifontein also helped me a lot because you see a lot of patients in that amount of time. So it’s good experience to get hands-on practice as well. So just to advise third-year students of outreaches you can go to …. (SV7) *Peer learning* • We were placed together in pairs for the block week. That was really helpful to have another student there. (SIII1) • [B]efore we went into the clinic on our own, we were paired up with a fourth year or a final year … to be able to observe someone before going in, I think you could help. (SIV2) • What also helps in clinics was peer teaching. Like when you shadow, like the fourth and fifth years—I felt like that really helped. Because then the fifth year can teach you stuff that the lecturer can’t. (SV2) *Case reviews* • [T]he same concept can be applied with the fifth years because it’s nice just to quickly—we know this student has a diabetic patient for instance. This was not supposed to be done, so we are going around it and now okay—‘[E]veryone needs to read up on it, and we have a discussion session’. Then you find of [*sic*] some of the students that feel they don’t want so speak out [and] gets [*sic*] the answers from someone else that is confident enough to speak up about it. That just makes it a bit easier as well, instead of you going to the clinic and the supervisors are hammering you the whole time, asking you this question, asking you that question …. (SV7) Feedback • … where you get some sort of feedback to say, ‘Okay, this is where you went wrong. I would have given you this mark if you had done so and so’. (SIII1) • [S]he gave me a mark. … the mark was okay, but I’m trying to say like it actually made me see that there’s room for improvement. So, I didn’t take the mark that I got as you know, I deserve more. I thought I deserved more but okay, obviously, I’m still studying … and definitely on the block course, I don’t think it’s a good thing to give us marks. But after the block course, it is kind of important to get marked and to also gauge where you’re at, and we could improve. And it also makes you kind of critically look at what you did and how you can do better next time. (SIII3) Visual technologies • [T]utorial videos are my thing, especially now we’re into technology and you have it. So now, instead of referring you to that lecture, you can refer to that video. Just take a very good video of the entire thing then audio and documents are immediately available. So there’s no … you don’t have to go and sit and wonder, ‘Okay, what did he do back then?’ (SV6)
	Clinical teachers’ recommendations to improve teaching the skill of tooth extraction: Teaching and learning strategies - ✓ Scaffolding ✓ Peer learning ✓ Visual technology	Scaffolding First time being introduced to this clinical setup. So, bombarding them is very overwhelming. So you don’t want to scare them off in a way; you want to ease them into a programme. So they can eventually, within the fourth year, maybe have a little bit more, how can I say?—extra things added onto, onto the already hectic; they always say [that] to a student. They also say ‘Doctor’s hectic’. That’s a term that was used to us. So, and then, they always feel overwhelmed. They sweat, and they feel the pressure when they enter the clinic. So you want to create an environment for them eventually where they feel comfortable, and they need to trust you. So, the gain of trust is also important. I agree with what they said; it’s very important for students to physically be able to process or do the instrument in the hand and physically seeing and touching things. Because to just show videos and just see lectures or attend lectures is not always necessarily beneficial to them, especially when you get different types of students. (CT3) Peer learning • [A]s a student, you feel scared … I’m not going to knock on the doctor’s door to ask her … so maybe if you have a peer facilitator that’s available to the students … they [the students] know they can go to that person and then the peer facilitator can be the connection to the doctors. (CT9) Visual technology • I think that the blended approach is definitely more important for students now than the didactic approach that we were given many years ago … [W]e found that blended techniques definitely helped students visualise and also contextualise what they needed. So, I definitely felt the videos were appropriate in terms of what they were saying. Maybe the manner in which the videos were presented may need to be relooked, be more relevant to a South African student. (CT7) • I’m gonna say the videos for me is the most important part. I mean, I myself am a visual learner. (CT7)
	Clinical teachers’ recommendations to improve teaching the skill of tooth extraction: Curriculum issues - ✓ Reducing the didactic teaching	• So what I can remember is that there’s a lot of lecture time where this is actually supposed to be a more of a clinical thing. So, I think techniques and instrumentation, stuff like that, that’s more important than sitting there repeating stuff that’s being said in, in different subjects like in pharmacology, etc. (CT8)
**DELIVERY/DESTINY *(Sustaining)***		
**Theme**	**Sub-Theme**	**Quotations (Response)**
Integration of knowledge and skills	The layout of the exodontia course is well planned.	• There’s nothing I would change about the block; it was really lovely. (SIII1) • [I]t was well balanced. [theory and practice] (SIV4) • It was all straightforward and precise and explicit. (SIV2)
Challenges associated	Reliance on teachers - ✓ Ownership of learning	• [T]his is my personal opinion that students, you don’t want to baby them. [B]ut I do feel sometimes … they are being babied up to a point where they say ‘its hectic’ or you need to ease them in. But I personally don’t feel that the pace was hectic; I think the pace was okay … there’s going to have to be a transition at some point. So they just need to learn to also adapt themselves, which I think students struggle to do personally. You know, we as clinicians or as teachers, yes, we can track from our side, but at the end of the day, they also need to take ownership and realise … this is a programme that I’m in and it’s going to, it’s going to be a little bit hectic at times. (CT9) • Students take chances. They know they’re gonna have to do, repeat that assessment until they pass. So for them, it’s a joke at the end of the day. They have that attitude of, ‘I don’t care, I’m here; my supervisor won’t fail me. I have multiple chances to pass’. (CT9)

## Summary and discussion of the findings

The use of the AI framework allowed for a comprehensive presentation of the findings pertaining to the clinical skills development component of the traditional course. The phases of the framework are not necessarily explicit, and possibly due to the generative nature of the process, crossover of the themes and sub-themes resulted. The authors mapped the information pertaining to the clinical skills component from the master table of findings (Table 3) to the various phases of the AI framework (Table 4). The phases are intertwined and closely linked (Fig 1). The Discovery phase highlighted the areas of skills development that were positively perceived by the research participants. The Dream phase reflected the views of what participants envisioned as potential positive contributors to the course, while the Design phase made suggestions as to how the recommendations for improvement could be incorporated into the new course. Lastly, the Delivery or Destiny phase concerned strategies for sustaining the changes of a revised course.

### Discovery

The most fundamental finding from the research was that the course, in its current form, met the basic requirements for teaching the skill of tooth extraction. This was the view expressed by all three of the research samples. The themes that identified aspects of the course that worked well included *Integration of knowledge and skills* and *Block course structure*. The sub-themes provided a more detailed view of the strengths of the course. The integration of the course content, didactic teaching, course presentation (layout), complemented by the incorporation of clinical practice and chairside teaching, contributed to the success of the course, as evidenced by the emerging sub-theme *Student confidence* [11]. Participant quotations or responses supportive of the Discovery phase are presented in Table 4. One of the contributors to the success of the course was the role of the CTs. The sub-theme, *Clinical teachers assist with consolidating theory and practice*, was a key feature in demonstrating the alignment of theory and the practical outcome. The role of the CT is instrumental in integrating theory and clinical practice is critical in the training of dentistry students. Clinical teachers play an important role in facilitating competence development [40–42]. Further findings of this research identify various other roles from being pastoral in nature to being content specialists or demonstrators and clinical chairside teachers. This highlights the importance of investing in CTs by providing the necessary tools and support to allow them to transition from practitioners to CTs. Approximately 80% of the participants in the CT sample spent a substantial proportion of their time as clinician practitioners in private practice. According to Davies et al. (2013), CTs with private practice experience contribute significantly to student clinical training because they possess a wealth of experience, knowledge, and expertise in the very area for which students are being prepared [43]. A wealth of information on CTs, extending from CT attributes, roles of CTs, and tools to enhance clinical teaching are available in the literature [14, 40, 44].

### Dream and design

The Dream phase reflects the results of the theme: Recommendation for improvement. Contained within this theme are the results of the sub-themes, which reflect the students’ views of the strategies that could improve the teaching of the tooth extraction skill, and the CTs views for improving the teaching (Table 4). The themes *Recommendation for improvement* and *Challenges associated* are forthcoming in the Design phase. Due to the inter-relationship of the phases of the AI framework, and the themes forthcoming from the data, we will discuss these in one section so as to ensure a coherence.

The student sample had strong views on the timing of the block course, and the use of elevators. These two issues were considered curriculum issues as it related to the content and outcomes of the course, and the shift of a unit within the module. As a recommendation to improve the teaching, the CT sample proposed a reduction in the amount of didactic teaching within the course. Other recommendations for the improvement of the course, put forth by the student sample and the CT sample, related to teaching and learning strategies.

The student sample expressed a strong desire to introduce the tooth extraction procedure earlier within the dentistry programme. The sentiment was that the additional time gained by shifting the course a semester earlier would allow them more time to gain proficiency in the procedure before exiting the programme, This is expressed by the quotation, “*If they can just move it to the beginning of third year … Basically*, *by the time we get to final year*, *you don’t really need a supervisor; they’re just supervising and checking tiny things”* (SIV4). Coupled with the earlier shift, students wanted to be taught the complete range of simple tooth extraction techniques ranging from forceps removal to that of elevators/luxators.

The use of elevators/luxators on patients versus its mere introduction and mention of its indications, was suggested as beneficial by students in their fourth and fifth years of study. The senior students felt that the staggered approach to the use of dental forceps and elevators/luxators hindered the development of the skill and also expressed the view that the use of elevators/luxators in the tooth extraction procedure was underutilised. A recent study at a UK dental school reported the use of instructional videos for increasing the self-confidence of dental students with the use of luxators [45]. Although the staggered approach may allow for scaffolding of the procedure and a subsequent reduction in the stress and anxiety experienced by students (quotation on Table 4) in the three-and-a-half-day course, the course coordinator may have to consider extending the duration of the course and amend the outcomes of the course to include elevators/luxators for the intra-alveolar (simple tooth extraction) procedure. The combination of forceps and elevators/luxators could potentially result in a less traumatic extraction with less bone and soft tissue destruction [46]. A recent study at a UK dental school reported the use of instructional videos for increasing self-confidence of dental students with luxator use [45]. Since the short duration of the course was a challenge for all samples, an extended course may permit for the inclusion of all instruments in the tooth extraction procedure. Inclusion of the physics forceps for removal of grossly decayed teeth [47] may then also be an option, so too an exposure to various other exodontia techniques [48]. Regarding the sub-theme regarding CTs recommendations for improvement, a suggestion was made to reduce the amount of didactic teaching which took place during the block course. CT8:

So what I can remember is that there’s a lot of lecture time where this is actually supposed to be a more of a clinical thing. So, I think techniques and instrumentation, stuff like that, that’s more important than sitting there repeating stuff that’s being said in, in different subjects like in pharmacology, etc.

Generally, block courses presented at the school are of a practical nature as opposed to being theoretical. The general feedback from the student sample regarding the course as a whole indicated that there was an adequate blend of didactics and hands-on practice. The response from one student, however, suggested that the course should have an even higher ratio of clinical practice to didactic teaching. A distinction is made between non-clinical modules such as ‘pharmacology’ that do not have a psychomotor component. This response is echoed by other sub-themes regarding time constraints. A concerted effort in prioritising clinical exposure in the future should be made. A consequence of the COVID-19 pandemic has been the exposure and equipping of faculty to more teaching technologies [49], which could assist with the delivery of content in a more exciting fashion and within a reduced time than the traditional face-to-face lecture time. Moreover, a greater reduction of didactic teaching should be considered with more infusion of blended-learning activities to teach content [50].

A number of teaching and learning strategies aimed at improving clinical skills development was raised by the student and CT participants. There appeared to be some overlap in these, which can be viewed in Table 4. The strategies identified to support skills development were debriefing, access to a skills laboratory or simulation laboratory, community-based learning, peer learning, case reviews during clinical practice, feedback, and visual technology.

Debriefing was a strategy that was suggested to increase the learning opportunities for students. Participant SV3 felt that when an unusual clinical situation arose (e.g. a medical emergency), it should be used as a teaching opportunity or a debriefing session for the benefit of the entire rostered group of students and not only for the student managing the patient. In addition, a debriefing session at the time of the incident may prevent possible long-lasting trauma for the novice clinician. Debriefing is an activity that has loosely been defined by Nocera and Merrit (2017) as a meeting for the purpose of review and discussion of team performance, education, identification of errors, emotional response and support, and the development of plans for the future [51]. Although common in the military, aviation, and other industries that are prone to stressful situations, debriefing has also been used in educational settings. It may be employed to support student learning by providing the opportunity for discussion and peer learning in addition to managing traumatic incidents [51, 52]. A quote reflecting the proposed benefits of debriefing is shared by participant SV3:

[I] really do think it’s because of that episode I had with the syncope patient, that could just, like deflated my confidence a whole lot. I’m not saying I’m a needy person, but if the supervisor/someone told me, you know, it’s gonna be fine, … maybe I just had the wrong supervisor on the wrong day, but just that debriefing, or just to let you know that it’s normal, it’s gonna happen and you’re only gonna grow from here…

The debriefing activity not only supports experiential learning, but is also used as a tool to reach consensus on the teaching content of CTs who teach in teams. Qualitative studies, literature reviews, and expert opinion papers have identified several characteristics for successful debriefing; these include structure, positive faculty demeanour, cueing questions, debriefing immediately after simulation, honest forthright feedback on performance, a safe learning environment, and adequate time to debrief learners [53].

The *clinical skills or simulation laboratory* is used in medical education for students to train in a simulated clinical environment. This development became necessary when the access to patients for clinical teaching and training was reduced for a number of reasons including an increase in student numbers and an increase in litigious claims. Whilst the skills laboratory is not conducive for teaching of exodontia in its current state, a skills laboratory allows for an increase in opportunity for students to view, to participate in demonstrations (“*[T]utorial videos coming from the staff members giving the block would be helpful on the chair positioning specifically because people are still doing it wrong even now”* (SV6), and to handle real instruments such as extraction forceps. The opportunities for practising clinical procedures in a simulated clinical environment with the correct instrumentation act as a form of *scaffolding*. Students are able to practise *patient and operator positions* and orientate themselves in terms of anatomical structures in the mouth in a low-risk environment. This helps in gaining confidence and reducing the stress that students endure when engaging with an invasive procedure such as the removal of a tooth. Less-stressed students may engage more confidently on the clinical training platform, enhancing their learning and ultimately resulting in improved patient outcomes. Owing to resource constraints, the traditional block course did not take place in a clinical skills laboratory equipped for oral surgery procedures. The suggestion to incorporate a clinical skills or simulation laboratory into the course in the future requires significant planning. The design and construction of a clinical skills laboratory requires resources in terms of finance and space together with buy-in from the relevant authorities.

*Community-based learning* or education, also referred to as ‘community outreach’, was viewed very favourably by the samples comprising students and DPs and was considered an opportunity to improve and accelerate clinical skills and to serve as an introduction to the *‘real world’*. The practice of working in communities provides insight into the inner workings of communities, including their culture, health practices, and challenges. According to DP5:

Comm-serve [community service], but I think it was in final year. We didn’t do outreach programmes, and I was part of the group that went to Gugulethu clinic. A group of us went there, and we got introduced to the real world, the real part of dentistry, and our clinical patient… was the government. And, you know, [you] see all those patients outside and you having a certain timeframe to complete it, you learn to work really well, and you learn to gain that confidence.

A concern raised by the CT sample was that the content presented in the course was too expansive and was not focused specifically on skills acquisition. The content pertaining to the use of drugs within Oral Surgery and prescription writing was beyond the parameters of the course and this possibly distracted the student from the task of skills acquisition. It was suggested that the time spent on delivering this content be used in developing the psychomotor skill by increasing the time spent on clinical practice.

Peer learning in medical education has become increasingly popular in part due to the increase in the number of medical students. Its benefits are well established in the literature [54, 55]. There are many permutations of how peer learning can occur. For the peer learner, ‘identifying with peers’ and ‘ease of communication’ appeared to be important factors for determining whether students would seek advice from peers as opposed to CTs. Similar learner styles and reduced stress and embarrassment were noted as reasons for these. Sharing of information was the most common reason for peer-to-peer interaction. The clinical competence modelled by senior students appeared to instil a feeling of confidence in junior students’ own potential. In addition, the students enjoyed being taught ‘shortcuts’ and ‘tricks’ by the senior students. For the peer tutor, the opportunity of imparting knowledge was empowering and allowed for the reinforcement of their own knowledge. The concept of peer learning was highlighted as a tool to support student learning, and it was recommended that its inclusion should be formalised in future courses. An added benefit is that the practice has been shown to foster the core competencies of the profession in student tutors [56].

Feedback is an important contributor to learning and forms part of the experiential learning cycle. SIII1 shared this view:

… where you get some sort of feedback to say, ‘Okay, this is where you went wrong. I would have given you this mark if you had done so and so’.

From the above response, there appears to be a mis-interpretation or misunderstanding between the provision of feedback and the link between assessment and feedback. Shrivastava et al (2022) indicate that faculty find it a challenge to provide feedback to students [57]. Many different reasons for this were provided. For this research, students felt that both assessment and feedback was necessary as a learning strategy. They did however feel that only feedback on their clinical procedures was necessary during the block course week and that assessment of the clinical activity combined with the feedback was of value as they progressed through the programme.

The benefits and value of technology (*visual technology*) in supporting learning was strongly supported in the research. Although a blended learning approach has been in use within the module, alternative applications of technology were described. The use of visual technology in the form of videos was found to be the most useful form of clinical teaching [45] apart from demonstrations and clinical practice on patients (experiential learning). Although CT7 mainly focused on the value of audio-visual technology, he also drew attention to the fact that the educational videos used in the course are developed for the USA market and that there may be benefit in developing video content that is more appropriate to the diverse South African context in terms of language, dental jargon, and instrumentation. The standardisation of terminology from the use of video-clips to the clinical platform will allow for the reinforcement of the concepts being taught. Furthermore, students would be more familiar with the South African jargon and would better identify with the terminology and its meaning. This could be a step in the direction of decolonising dental education at the institution. Globally, the trend towards decolonising higher education has gained momentum, with the medical fraternity subscribing a little later [58].

The theme *Challenges associated with the traditional block course* identified features that are amenable to redesign. The sub-theme, *Student stress and anxiety*, has been identified in dentistry literature. In the context of this research, the issue of anxiety was raised by a third-year student and a CT. The student perspective was that anxiety was reduced through knowing that the CT was there to assist if needed. The view of the CT was that more emphasis on chairside teaching should occur with the novice students than on ascribing a score for the clinical session because the assessment contributed to student anxiety and was detrimental to skills development at the ‘beginner/novice’ stage. Other challenges that were highlighted in this phase concerned logistical issues such as the size of the clinical rotation groups and the duration of the block course. The large numbers of students in the rotation groups negatively affected student learning, as demonstrated in the comment that visualising demonstrations centred on the small area of the mouth was difficult. Clinical teachers were of the opinion that the duration of the block course should be extended in order to increase the opportunity for engagement between the student and the clinical teacher on the training platform. Their view was that the clinical teacher to student ratio was too high and hence, an increase in time would increase the opportunity for individual attention. These concerns were supported by quotations such as, “*There were about 10 to 12 of us in one group*, *and obviously*, *the mouth is such a small area that we’re placed around him to kind of engage and see*. *You know*, *it’s kind of difficult”* (SIII1), and

*The third-year students are very fresh*. *So you basically hold their hand the whole way from beginning to end as soon as they walk into the cubicle until they leave*. *So*, *and that makes it difficult when it’s a big class because they need the individual attention*. (CT8)

An increase in the number of students within a group rotating through the course appeared to affect student learning negatively. Smaller group sizes were favoured by both students and CTs. A smaller number of students per group improved the student to clinical teacher ratio. This appeared to favour interaction between student and faculty member. The potential for opportunity for students to engage with faculty members increases when the student number decreases. In addition, demonstrations can be viewed more easily by students when there are fewer students in the group. In terms of redesigning (co-constructing) the course, a reduction in the number of students in the course rotation will have to be considered. Human resources, physical infrastructure, and time availability will also have to be taken into account. The sub-theme ‘*Block course duration*’ encompasses the host of challenges that were encountered, particularly by the CT sample. The results suggest that better clinical teaching with the consequent student learning could take place if more time were spent on individual students. The following were viewed as hindrances to optimal clinical skills teaching and training: i) high student to clinical teacher ratio; ii) inadequate time per clinical session to teach; iii) disproportionate focus on clinical service delivery versus clinical chairside teaching; iv) an over teach may be taking place, breaching the objectives of the course; and v) too much emphasis on the clinical assessment (assessment of learning) of the junior student, thus negatively affecting student learning. Clinical teachers expressed that increased opportunity to engage with students on an individual basis would allow them to impart skills teaching to novice clinicians. Contemporaneously, the students sample expressed an increase in the duration of the course to allow them more time for practice. With the luxury of having adequate patients requiring treatment (opportunity for practice), increasing the duration of the block course or recommending block teaching to the institution may address the issue of time constraints and aid in meaningful clinical teaching and learning. Of the recommendations for improvement of the block course suggested by the student sample, teaching and learning strategies such as community-based education, peer learning, feedback, case reviews during clinical practice, and visual technologies featured prominently.

### Delivery/Destiny

This phase is represented by the themes: *Integration of knowledge and skills* and *Challenges associated*.

The sub-theme, the layout of the course is well planned, as evident by the quotation:

There is nothing I would change about the block, it was lovely (SIII1)

Because the overall view of the traditional block course was positive, the retention of the aspects which were both well received and well perceived by the participants are important when developing the revised course. A consideration in addressing the sub-theme, *Reliance on teachers*, may require the exploration of strategies which foster independent, adult learning. Self-directed learning (SDL) is an example of a strategy that can be explored in an attempt to address the lack of ownership for learning by some students, as expressed by the CTs in this research. Self-directed learning has been suggested as a promising methodology for lifelong learning in medicine [44]. This strategy is closely linked to the principles associated with adult learning. According to Knowles (1980), adults learn differently from children and, therefore, they introduced the term andragogy as opposed to pedagogy to describe learning in adults [59]. Additionally, the incorporation of teaching and learning strategies such as flipped classroom may also contribute to an environment where students are primed for more independent learning [50]. The graduate attributes aspired to at the institution may have to be more explicitly embedded to achieve a stronger measure of learning ownership. The graduate attributes are, among others, the encouragement of active citizenry and life-long learning.

The number of student participants in the research can viewed as a limitation. An increase in the amount of student participation may add further depth to the themes generated by the data. Students learning are diverse and there may possibly be an outlier in terms of student learner styles which has not been included in the research. An analysis more specific to the elements of the curriculum may have resulted in themes for pointed to these elements.

## Conclusions

The evaluation process brought to light the strengths and concerns of the course from the perspectives of three stakeholder groups. Moving forward, it would be necessary to interrogate and cross-match the views of each sample on specific focus areas of the curriculum. It is important to note that the proposed changes to the curriculum would have a knock-on effect across the elements. Because the tooth extraction procedure is fairly invasive, it is worth investing in instructional methods and learning strategies which support the journey from novice to competent in particular those suggested by the student and CT samples. The evaluation of the course clearly describes a continuum of learning of the tooth extraction procedure whereby the block course stems the starting point for skills development, with the continuation of clinical learning throughout the fourth and fifth years providing the opportunity to gain proficiency and ultimately reaching mastery if the correct instructional methods being applied. Although the research made use of only the subjective, personal views and experiences of the stakeholders, the nature of the qualitative data collection approach allowed for the retrieval of rich data that were able to inform the status of the course and provide a basis for its redesign. The information that was obtained can be considered when planning and developing the course curriculum to promote effective clinical rotations and student learning. There have been significant technological advances in both dental extraction techniques and in the education sphere. Adding to this, the COVID-19 pandemic has opened up a space for online activities which supports a student-centred, adult learning approach. A convergence of these three factors could potentially result in a revised course which meets the needs of the majority of diverse students. The findings of this research provide a baseline allowing for comparison of the course with similar oral surgery modules at other dental schools. Further, the results of this research augment the literature currently available on best practice for exodontia skills acquisition and provide important baseline information for the planning and redesign of related courses.
